# The Effects of Bee Venom Acupuncture on the Central Nervous System and Muscle in an Animal hSOD1^G93A^ Mutant

**DOI:** 10.3390/toxins7030846

**Published:** 2015-03-13

**Authors:** MuDan Cai, Sun-Mi Choi, Eun Jin Yang

**Affiliations:** 1 Department of KM Fundamental Research, Korea Institute of Oriental Medicine, 483 Expo-ro, Daejeon, Yuseong-gu 305-811, Korea; E-Mail: mudan126@kiom.re.kr; 2 Executive Director of R&D, Korea Institute of Oriental Medicine, 483 Expo-ro, Daejeon, Yuseong-gu 305-811, Korea; E-Mail: smchoi@kiom.re.kr

**Keywords:** amyotrophic lateral sclerosis, bee venom acupuncture, acupoint, central nervous system

## Abstract

Amyotrophic lateral sclerosis (ALS) is caused by the degeneration of lower and upper motor neurons, leading to muscle paralysis and respiratory failure. However, there is no effective drug or therapy to treat ALS. Complementary and alternative medicine (CAM), including acupuncture, pharmacopuncture, herbal medicine, and massage is popular due to the significant limitations of conventional therapy. Bee venom acupuncture (BVA), also known as one of pharmacopunctures, has been used in Oriental medicine to treat inflammatory diseases. The purpose of this study is to investigate the effect of BVA on the central nervous system (CNS) and muscle in symptomatic hSOD1^G93A^ transgenic mice, an animal model of ALS. Our findings show that BVA at ST36 enhanced motor function and decreased motor neuron death in the spinal cord compared to that observed in hSOD1^G93A^ transgenic mice injected intraperitoneally (i.p.) with BV. Furthermore, BV treatment at ST36 eliminated signaling downstream of inflammatory proteins such as TLR4 in the spinal cords of symptomatic hSOD1^G93A^ transgenic mice. However, i.p. treatment with BV reduced the levels of TNF-α and Bcl-2 expression in the muscle hSOD1^G93A^ transgenic mice. Taken together, our findings suggest that BV pharmacopuncture into certain acupoints may act as a chemical stimulant to activate those acupoints and subsequently engage the endogenous immune modulatory system in the CNS in an animal model of ALS.

## 1. Introduction

Amyotrophic lateral sclerosis (ALS) is caused by the selective and progressive loss of motor neurons, leading to irreversible paralysis; speech, swallowing and respiratory malfunction; and eventually the death of the affected individual after a rapid disease course. ALS is mostly sporadic; 90% of ALS cases occur without a family history of the disease. Mutations in the gene encoding superoxide dismutase-1 (SOD1) cause 15%–20% of familial ALS (fALS) cases, corresponding to 1%–2% of all ALS cases [1]. Recent clinical and electrophysiological data show that the human SOD1-G93A phenotype closely resembles sporadic ALS, indicating comparable disease pathology [2].

Neuroinflammation by glial cells is a normal and necessary process to support neuron cell survival. However, excess neuroinflammation is detrimental, especially in multiple sclerosis (MS), ALS, various types of dementia, Huntington’s disease, and other diseases [3,4]. In ALS, non-neuronal cells, such as astrocytes and microglia, release neurotoxic factors and induce neuroinflammatory events causing motor neuron death [5,6]. Several studies related to neuroinflammation by microglia and astrocytes have reported that inflammation is fundamental to the pathogenesis of ALS and have suggested that anti-inflammatory drugs may play an important role in treating ALS patients [7,8,9]. Anti-inflammatory agents including celastrol, thalidomide, lenalidomide, NDGA, and pioglitazone have delayed the progress of disease in ALS animal models but require further evaluation in clinical studies [10].

Bee venom (BV) or apitoxin is extracted from honeybees and is commonly used in Oriental medicine. BV therapy is used for anticoagulant and anti-inflammation treatments in rheumatism and joint diseases [11]. BV treatment has been shown to reduce pain in patients with chronic rheumatoid arthritis or osteoarthritis [12]. In addition, recent papers have reported that BV treatment has a neuroprotective role against neurodegenerative diseases such as Parkinson’s disease (PD) and ALS [13,14].

Pharmacopuncture, injection to acupoints with pharmacological medication or herbal medicine, is a new acupuncture therapy widely available in Korea and China. However, its effectiveness remains unclear. Pharmacopuncture has been used for cancer-related symptoms [15] and an Australian study reported that it induces higher de-qi sensation compared to traditional acupuncture, which may indicate that pharmacopuncture could provide stronger clinical responses than traditional acupuncture [16].

Pharmacopuncture with BV, referred to as bee venom acupuncture (BVA) is applied at specific acupoints to enhance the effect of BV and acupuncture in Korea. Joksamni (ST36) acupoint is known to mediate anti-inflammatory effects [17] and we have demonstrated that Joksamni (ST36) acupoint stimulation enhanced anti-neuroinflammation in symptomatic hSOD1^G93A^ transgenic mice [18]. The purpose of this study is to investigate BVA at ST36 to identify the effects of acupuncture and BV in the CNS and muscle in symptomatic hSOD1^G93A^ transgenic mice. For this study, we established four groups of mice: Non-Tg mice treated with saline acupuncture at ST36 (Non-Tg), hSOD1^G93A^ mice treated with saline (CON) or BVA at ST36 (ST36), and hSOD1^G93A^ mice injected intraperitoneally (i.p.) with BV (IP).

We found that BV treatment significantly improved walking function compared to BV-i.p.-injected hSOD1^G93A^ mice and age-matched hSOD1^G93A^ mice treated with saline acupuncture at ST36. In addition, BVA at ST36 reduced the levels of neuroinflammatory proteins such as Toll-like receptor 4 (TLR4), CD14, and Tumor Necrosis Factor-alpha (TNF-α) in the spinal cord compared with saline acupuncture at ST36-treated hSOD1^G93A^ mice, but BV-i.p. injection in symptomatic hSOD1^G93A^ mice did not. Furthermore, we detected that the nuclear abnormality in the quadriceps femoris muscle was significantly reduced by BVA compared with saline acupuncture at ST36 in ALS mice but was not affected by i.p. injection of BV in hSOD1^G93A^ mice. These findings suggest that BVA at ST36 can be more effective than either ST36 stimulation or BV injection alone in reducing neuroinflammation in the spinal cord of hSOD1^G93A^ transgenic mice.

## 2. Results

### 2.1. BV Treatment Improves Motor Functions in Symptomatic hSOD1^G93A^ Mice

**Figure 1:**
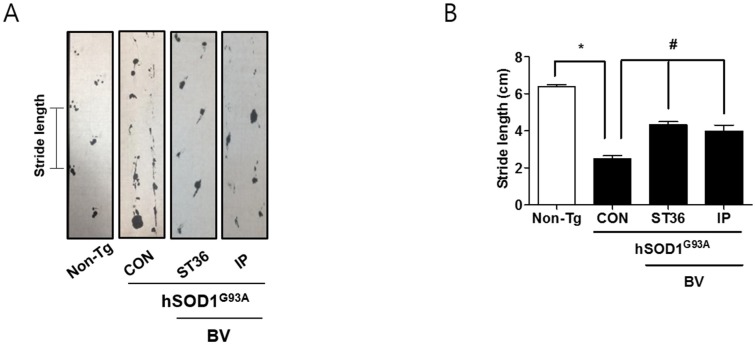
Bee venom (BV) treatment improves stride length in footprints test of hSOD1^G93A^ transgenic mice. Comparison of footprints between groups (**A**). Non-transgenic (Non-Tg) mice are B6SJL mice, and hSOD1^G93A^ transgenic mice were divided into three groups: saline acupuncture at ST36 (CON); BVA at ST36 (ST36); i.p. administration of BV (IP). Quantification of stride length from each group (**B**). (*n* = 9–10/group) Each bar represents the group mean ± SEM (*, # *p* < 0.05).

To investigate the effect of BV treatment at ST36 or i.p. injection of BV on motor function of symptomatic hSOD1^G93A^ transgenic mice, we conducted the footprint test as a behavioral test. As shown in Figure 1, symptomatic hSOD1^G93A^ transgenic mice (CON group) showed stride lengths of 2.64 ± 0.19 cm and reduced by 2.4-fold compared to that of Non-Tg. However, both BVA at ST36 (ST36 group) and IP group increased stride length by 1.6-fold, and 1.5-fold, respectively, compared to that of saline acupuncture at ST36 (Figure 1B, *, # *p* < 0.05). This result suggests that BV treatment, regardless of the method of administration, could effectively improve motor function in hSOD1^G93A^ transgenic mice.

### 2.2. BV Treatment Reduces Motor Neuron Cell Death in Symptomatic hSOD1^G93A^ Mice

**Figure 2:**
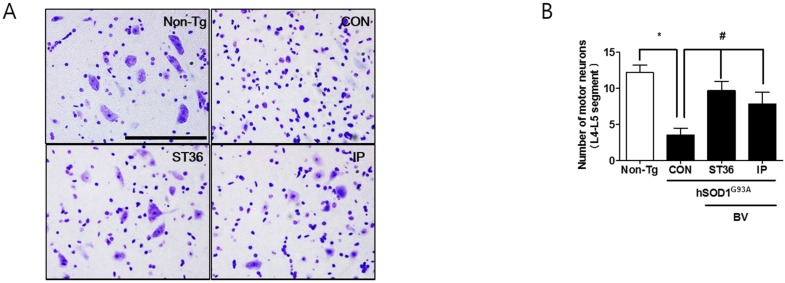
BV treatment alleviates the decrease of motor neurons in hSOD1^G93A^ transgenic mice. Representative photographs of Nissl staining in the ventral horn of the spinal cord of hSOD1^G93A^ transgenic mice (**A**). Quantification of the motor neurons in the L4-L5 segment of the spinal cord in hSOD1^G93A^ transgenic mice (*n* = 4/group) (**B**). Each bar represents the group mean ± SEM (*, # *p* < 0.05). Scale bars = 200 µm.

Based on the result of Figure 1, we determined whether different BV treatment affect motor neuron death in the spinal cord of symptomatic hSOD1^G93A^ mice. Based on Nissl staining, the numbers of motor neurons in the spinal cord of symptomatic hSOD1^G93A^ transgenic mice (CON group) were decreased by 3.5-fold compared to that of Non-Tg (Figure 2A,B, *, # *p* < 0.05). Both BVA at ST36 and i.p. administration of BV were associated with more than twice as many neurons as saline acupuncture at ST36 (Figure 2B). No statistical significance was found between BVA at ST36 and i.p. administration of BV, demonstrating that BV has a neuroprotective role in an animal model of ALS, regardless of the treatment method.

### 2.3. BVA at ST36 Augments Anti-Neuroinflammation

**Figure 3:**
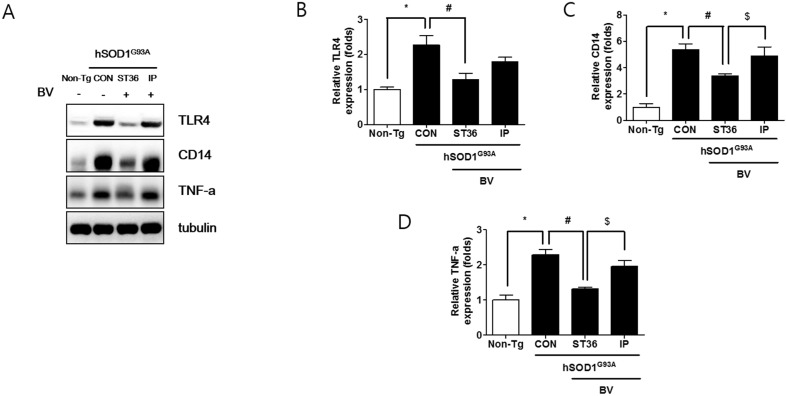
BVA at ST36 eliminates Toll-like receptor 4 (TLR4) signaling-related inflammatory proteins in the spinal cord of ALS mice. Representative images of Western blots for TLR4, CD14, and TNF-α (**A**) (*n* = 5–6/group). Tubulin was used as a loading control. Quantification of immunoblots (**B**–**D**). Each bar represents the group mean ± SEM (*, #, $ *p* < 0.05).

To determine the mechanism underlying the effects of BVA at ST36, we studied the effects of BV treatment on neuroinflammation in the spinal cord of symptomatic hSOD1^G93A^ transgenic mice. As expected, BVA at ST36 significantly reduced the levels of the inflammatory proteins TLR4, CD14, and TNF-α, by 1.8-fold, 1.6-fold and 1.7-fold, respectively, in the spinal cords of symptomatic hSOD1^G93A^ transgenic mice compared to those of Non-Tg (Figure 3A–D). However, i.p. administration of BV did not have a similar effect. This suggests that BVA at ST36 prevents motor neuron death by inhibiting neuroinflammation in the spinal cord of symptomatic ALS mice.

### 2.4. BVA at ST36 Prevents Muscle Atrophy in Symptomatic hSOD1^G93A^ Transgenic Mice

**Figure 4:**
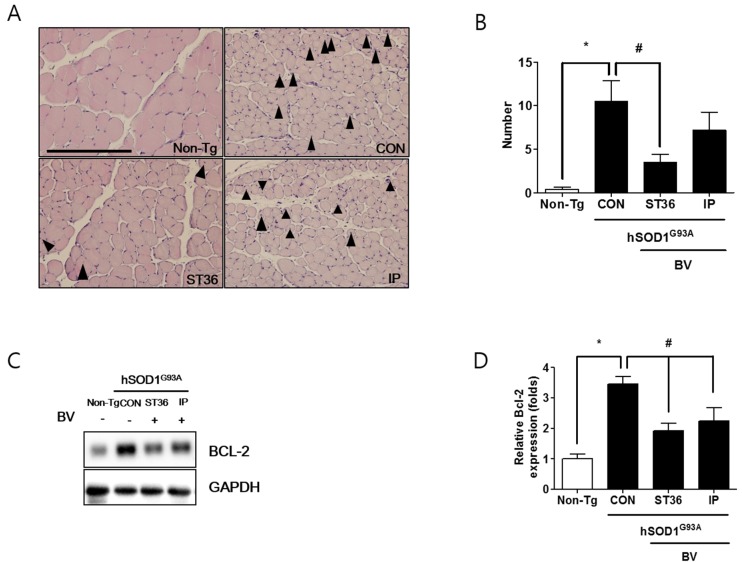
BVA at ST36 reduces nuclear abnormalities in fibers of the quadriceps femoris muscle from hSOD1^G93A^ transgenic mice, viewed in transverse sections. Hematoxylin and eosin staining of quadriceps of Non-Tg, hSOD1^G93A^ transgenic mice (CON), transgenic mice treated with BV at ST36 (ST36), transgenic mice treated with i.p. BV (IP). Arrowhead indicates fiber with abnormal nuclei (**A**); Quantification of mean numbers of abnormal nuclei in fibers of quadriceps femoris muscle of hSOD1^G93A^ mice (**B**); Graph, mean ± SEM, *n* = 5–6/group (*, # *p* < 0.05). Representative images of the expression of Bcl-2 in quadriceps femoris muscle from the Non-Tg, CON, ST36, and IP groups (**C**); GAPDH was used as a loading control. One representative experiment of three replicates is shown. Quantification of immunoblots (**D**); Each bar represents the group mean ± SEM, *n* = 3/group (* *p* < 0.05, # *p* < 0.05). Scale bars = 200 µm.

**Figure 5:**
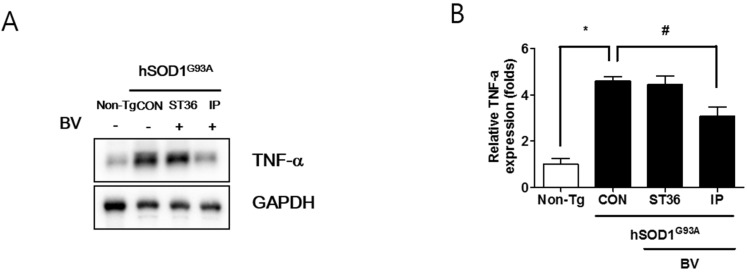
Intraperitoneal injection of BV reduced the levels of inflammatory proteins in quadriceps femoris muscle. Tissue lysates from the quadriceps femoris muscle from the Non-Tg, CON, ST36, and IP groups were immunoblotted with anti-TNF-α (**A**). GAPDH was used as a loading control. Quantification of immunoblots (**B**). Each bar represents the group mean ± SEM (*n* = 3/group) (*, # *p* < 0.05).

To evaluate the effect of BV treatment and the method of administration on the muscle pathology of hSOD1^G93A^ transgenic mice, we examined the effect of BV treatment on fibers of the quadriceps femoris muscle in the hind leg, a target of innervation by the spinal motor neurons counted. As shown in Figure 4A, the fiber diameter was smaller in hSOD1^G93A^ transgenic mice than in Non-Tg. In addition, myofibers containing abnormal nuclei were increased by 10-fold higher than compared to Non-Tg mice. Interestingly, BV treatment at ST36 reduced the number of abnormal nuclei to 3-fold than that of the control (Figure 4B). Intraperitoneal injection of BV reduced the fibers with abnormal nuclei compared to that of saline-treated hSOD1^G93A^ transgenic mice, but the decrease was not significant. To investigate whether BV treatment affected the mitochondrial function of myofibers, we examined the expression level of Bcl-2 protein in the quadriceps femoris muscle. As shown in Figure 4C, the expression level of Bcl-2 was increased by 3.4-fold in CON compared to Non-Tg. However, after BV treatment at ST36 and via i.p. injection, the level of Bcl-2 protein in the quadriceps femoris muscle was 1.8-fold, and 1.5 fold lower, respectively, compared to CON. These findings suggest that BV treatment could ameliorate the mitochondrial dysfunction and muscle structure defects observed in an animal model of ALS. To establish the factor for improvement in walking ability achieved by BV-injected i.p., we examined the expression level of a pro-inflammatory protein, TNF-α, in the quadriceps femoris muscle of hSOD1^G93A^ transgenic mice. As shown in Figure 5, the expression of TNF-α was increased by 4.6-fold in the quadriceps femoris muscle of symptomatic hSOD1^G93A^ mice. However, i.p. injection of BV reduced the level of TNF-α to only 3.1-fold higher than in CON. BVA at ST36 treatment did not reduce the expression level of TNF-α as much as i.p. injected BV in symptomatic hSOD1^G93A^ transgenic mice. Since BV causes inflammation in the skin [19], inflammatory protein such as TNF-α did not reduce in the muscle of BVA at ST36 even though BVA at ST36 induced anti-neuroinflammation in the spinal cord of hSOD1^G93A^ transgenic mice. These data suggest that BV injection i.p. can reduce inflammation in muscle even though it does not reduce neuroinflammation in the spinal cord of hSOD1^G93A^ mice.

## 3. Discussion

Amyotrophic lateral sclerosis (ALS) is a neurodegenerative disease that causes progressive degeneration of motor neurons in the motor cortex, brainstem, and spinal cord. Familial ALS (fALS), caused by genetic mutations in genes such as Cu^2+^/Zn^2+^ SOD-1, TAR DNA binding protein-43 (TDP-43), and GGGGCC repeat expansions in the C9orf72 locus, represents 5%–10% of total ALS cases, whereas sporadic ALS (sALS), which is unassociated with any known genetic mutations, represents approximately 90% of ALS cases. To date, the pathological mechanism of ALS remains unclear because ALS is a complex syndrome involving not only motor neurons but also astrocytes, microglia, oligodendrocytes, and muscle cells in both fALS and sALS cases. Because there is no effective treatment for ALS patients, research toward ALS therapy remains ongoing.

Complementary and alternative medicine (CAM), including acupuncture, pharmacoacupuncture, herbal medicine, and massage, is popular due to the significant limitations of conventional therapy. It has previously been reported that 40% of patients with ALS in Germany used CAM therapies, along with 54% of patients in the United Kingdom and an even higher percentage in Asia. Acupuncture is one of the most popular alternative therapies used by patients with ALS [20,21]. Pan *et al.* have reported that ALS patients in Shanghai used CAM therapies including acupuncture, vitamin E, and herbal medicine to reduce the side effects of Riluzole [22]. Wasner *et al*. surveyed the use of CAM by ALS patients and reported that acupuncture, homeopathy, naturopathy, and esoteric treatments were widely used [23].

BV contains several bioactive compounds including melittin, phospholipase A_2_, apamin and peptides [24]. BV therapy has been used in Oriental medicine, and BVA is considered to have distinct effects via acupuncture and herbal medicine. However, the mechanism underlying the synergy of BV with acupuncture remains to be identified, even though several papers have reported anti-nociceptive or anti-inflammatory effects of BV in pain, arthritis, rheumatoid diseases, cancer, and skin diseases [11,25]. In addition, recent reports have shown that BV treatment has anti-neuroinflammatory effects in neurodegenerative diseases such as PD and ALS [13,14]. Kim *et al.* showed that BV injection into ST36 increased the modulation of METH-induced hyperactivity and hyperthermia via the central α_2_-adrenergic activation compared to other acupoints such as SP9 and GB39 [26]. In an animal model of ALS, BVA protected against motor neuron death through an increase of anti-neuroinflammation in hSOD1^G93A^ transgenic mice [14]. However, it remains to be determined whether the effect of BVA at ST36 is a synergistic effect dependent on acupoint stimulation in combination with BV or is an effect of BV alone. This study tested hSOD1^G93A^ transgenic mice with saline treatment at ST36 for acupoint stimulation, BV treatment at ST36 for acupoint stimulation plus BV effect, and i.p. injection of BV for the effect of BV alone. Interestingly, we found that both BVA at ST36 and i.p. injection of BV enhanced motor function and decreased motor neuron death in the spinal cord compared to saline-treated at ST36 in hSOD1^G93A^ transgenic mice. Furthermore, BVA at ST36 eliminates signaling by inflammatory proteins such as TLR4 in the spinal cord of symptomatic hSOD1^G93A^ transgenic mice. These findings suggest that BV treatment could improve muscle function and reduce motor neuron death, delaying the progress of disease onset in hSOD1^G93A^ transgenic mice.

Studies of ALS have demonstrated functional abnormality and muscle pathology in both fALS and sALS cases [27,28]. In an animal model of ALS, the alteration of myogenesis by the aberrant expression of Pax7 and myogenic regulatory factors induces skeletal muscle defects [29]. Kaspar *et al.* have reported that IGF-1 overexpression in skeletal muscle increased survival and prevented motor neuron death in an animal model of ALS, although a clinical study using IGF-1 therapy did not demonstrate any improvement [30]. To investigate the effect of BV treatment on the quadriceps femoris muscle in the hind limb, we examined myoblast pathology using histochemical and biochemical approaches. BVA at ST36 significantly reduced the number of fibers with abnormal nuclei compared to symptomatic hSOD1^G93A^ mice treated with saline at ST36. In addition, i.p. injection of BV significantly reduced the expression of TNF-α, a pro-inflammatory factor, in the quadriceps femoris muscle of symptomatic hSOD1^G93A^ mice. Taken together, our findings suggest that BV pharmacopuncture into certain acupoints may act as a chemical stimulant that activates the acupoint and subsequently engages the endogenous immune modulatory system in the CNS of an ALS animal model. In addition, the selection of a specific acupoint may be a key factor in producing pharmacological effects of BV acupuncture in an ALS animal model.

Demonstrating the effect of BV on the muscle function requires the study of mitochondria in the muscle of hSOD1^G93A^ transgenic mice. In addition, the effects of BVA at other acupoints should be studied to demonstrate the mechanism underlying the combined effects of BV and acupuncture. Bee venom therapy should consider on sensitization because it has on the risk of anaphylaxis.

## 4. Materials and Methods

### 4.1. Animals

Human-SOD1 (hSOD1) G93A transgenic (Tg) mice were purchased from the Jackson Laboratory (Bar Harbor, ME, USA) and maintained as described previously [14]. All mice were allowed access to water and food *ad libitum* and were maintained under a constant temperature (21 ± 2 °C) and humidity (50% ± 10%) under a 12-h light/dark cycle (light on 07:00–19:00). All mice were handled in accordance with the animal care guidelines of the Korea Institute of Oriental Medicine. The 14-week-old transgenic mice were considered symptomatic; mice of this age were used for the study.

### 4.2. Bee Venom Treatment

Bee venom was purchased from Sigma (St. Louis, MO, USA) and diluted with saline. In this experimental paradigm, the mice were divided into four groups: non-transgenic mice treated with saline at ST36, Non-Tg, *n* = 9; hSOD1^G93A^ mice treated with saline at ST36, CON, *n* = 10; hSOD1^G93A^ mice treated with BVA at ST36, ST36, *n* = 10; and hSOD1^G93A^ mice injected i.p. with BV, IP, *n* = 10. The ST36 acupoint is based on the human acupoint landmark and a mouse anatomical reference [31]; it is anatomically located at 5 mm below and lateral to the anterior tubercle of the tibia. Bee venom was injected into 14-week-old (98-day-old) male hSOD1^G93A^ transgenic mice. The Non-Tg and CON groups were bilaterally injected (subcutaneously) with an equal volume of saline at the ST36 acupoint. BV treatment at 0.1 µg/g was performed three times per week for two weeks. The total amount of BV treatment at ST36-mice or IP-mice was 0.6 µg/g for two weeks.

### 4.3. Footprint Test

On the second day after the final treatment, we performed a footprint test. Footprints were used to estimate stride length and hind-base width, which reflects the extent of muscle loosening [32,33]. The mice crossed an illuminated alley, 70 cm in length, 12 cm in width, and 16 cm in height, before entering a dark box at the end. Their hindpaws were coated with nontoxic water-soluble ink, and the alley floor was covered with white paper. To obtain clearly visible footprints, at least three trials were conducted. The footprints were then scanned, and stride length was measured using Image J software (NIH, Bethesda, MD, USA).

### 4.4. Western Blot Analysis

Western blotting was conducted as previously described [34]. Mice were sacrificed immediately after the behavioral testing, and the spinal cords were isolated for Western blotting. The spinal cords and quadriceps femoris muscle were dissected and homogenized in RIPA buffer (50 mM·Tris-HCl, pH 7.4, 1% NP-40, 0.1% SDS, and 150 mM·NaCl) containing a protease inhibitor cocktail. Following homogenization, protein was quantified using the BCA assay. Samples of 20 µg protein were denatured with sodium dodecyl sulfate sampling buffer and then separated using SDS-PAGE, followed by transfer to a PVDF membrane. For the detection of target proteins, the membranes were blocked with 5% non-fat milk in TBS (50 mM Tris-HCl, pH 7.6, 150 mM·NaCl) and subsequently incubated overnight with various primary antibodies: anti-tubulin, anti-TLR4, anti-CD14, anti-TNF-α, anti- GAPDH or anti-Bcl-2. The blots were then probed with peroxidase-conjugated secondary antibodies (Santa Cruz Biotechnology, Santa Cruz, CA, USA) and visualized using enhanced chemiluminescence reagents (Amersham Pharmacia, Piscataway, NJ, USA). Protein bands were detected with the Fusion SL4-imaging system (Fusion, Eberhardzell, Germany). Quantification of the immunoblotting bands was conducted with Image J.

### 4.5. Tissue Preparation

After the behavioral testing, all mice were anesthetized with an intraperitoneal injection of pentobarbital and perfused with phosphate buffered saline (PBS). The quadriceps femoris muscle and spinal cord tissues were removed and fixed in 4% paraformaldehyde for 3 days at 4 °C until embedding. Briefly, the quadriceps muscle and lumbar 4–5 spinal cord were embedded in paraffin, and the prepared tissues were cut into transverse sections (5 μm thick) and mounted on glass slides. Before staining, sections were deparaffinized in xylene and rehydrated in a graded series of ethanol followed by dH_2_O.

### 4.6. Nissl Staining

The staining was used to evaluate the general neuronal morphology and demonstrate the loss of Nissl substances [35] and was performed as previously described [36]. Briefly, after deparaffinizing, the sections of spinal cord were oven-dried, stained with 0.1% cresyl violet, dehydrated through a graded ethanol series (70%, 80%, 90%, and 100% × 2), placed in xylene, and covered with a coverslip after the addition of Histomount media. To quantify the Nissl staining, the cells in the ventral horn of the spinal cord were counted using Image J (version 1.46j) by one researcher blind to the treatment groups. Cells meeting the following criteria were counted: (1) neurons located in the anterior horn ventral to the line tangential to the ventral tip of the central canal; and (2) neurons with a maximum diameter of 20 μm or more.

### 4.7. H&E Staining

After being deparaffinized, slices of quadriceps muscle were incubated in hematoxylin (Sigma, St. Louis, MO, USA) followed by incubation in eosin. Slices were mounted with Histomount medium (Sigma, St. Louis, MO, USA). Analysis of H&E staining was performed in a blinded test, and fibers with central nuclei were counted in randomly selected areas from each mouse. Three stained sections were counted per hSOD1^G93A^ mouse; tissue samples from 5 to 6 mices per group were stained.

### 4.8. Statistical Analysis

All data were analyzed using GraphPad Prism 5.0 (GraphPad Software, San Diego, CA, USA) and are presented as the mean ± SEM where indicated. The results of the behavioral test and the histological and Western blot analyses were analyzed using one-way analysis of variance (ANOVA) followed by the Bonferroni’s post-hoc tests for multiple comparisons. Statistical significance was set at *p* < 0.05.

## 5. Conclusions

In summary, our data show that BVA at ST36 and i.p. injection with BV enhanced motor function and decreased motor neuron death in the spinal cord compared to that observed in hSOD1^G93A^ transgenic mice treated saline. Furthermore, BV treatment at ST36 eliminated signaling downstream of inflammatory proteins such as TLR4 in the spinal cords of symptomatic hSOD1^G93A^ transgenic mice. However, i.p. treatment with BV reduced the levels of TNF-α and Bcl-2 expression in the muscle of hSOD1^G93A^ transgenic mice. Taken together, our findings suggest that BV pharmacopuncture into certain acupoints may act as a chemical stimulant to activate acupoints and subsequently engage the endogenous immune modulatory system in the CNS in an animal model of ALS.
